# Interfacial Characterization of a Conventional Glass-Ionomer Cement after Functioning for 1-year In Vivo

**DOI:** 10.3290/j.jad.b2916453

**Published:** 2022-04-13

**Authors:** Shuhei Hoshika, Kenichi Koshiro, Satoshi Inoue, Toru Tanaka, Hidehiko Sano, Sharanbir K. Sidhu

**Affiliations:** a Assistant Professor, Hokkaido University Graduate School of Dentistry, Division of Oral Health Science, Department of Restorative Dentistry, Sapporo, Japan. Performed experiments, wrote the manuscript.; b General Practitioner, Hokkaido University Graduate School of Dentistry, Division of Oral Health Science, Department of Restorative Dentistry, Sapporo, Japan. Performed the experiments, wrote the manuscript.; c Professor, Section of Clinical Education, Faculty of Dental Medicine, Hokkaido University, Sapporo, Japan. Contributed to discussion.; d Lecturer, Hokkaido University Graduate School of Dentistry, Division of Oral Health Science, Department of Restorative Dentistry, Sapporo, Japan. Performed the experiments.; e Professor, Hokkaido University Graduate School of Dentistry, Division of Oral Health Science, Department of Restorative Dentistry, Sapporo, Japan. Idea, proofread the manuscript.; f Reader/Honorary Consultant in Restorative Dentistry, Institute of Dentistry, Barts and The London School of Medicine and Dentistry, Queen Mary University of London, London, UK. Contributed to discussion, proofread the manuscript.

**Keywords:** adhesion to dentin, adhesive dentistry, adhesive interface, glass-ionomer cements, TEM

## Abstract

**Purpose:**

To morphologically evaluate the interface between a conventional glass-ionomer cement (GIC) and dentin one day after placement, as well as the changes at the interface after one year of aging/functioning in monkey teeth.

**Materials and Methods:**

On the buccal surfaces of seven intact teeth in each of two monkeys, shallow class V cavities were prepared, which were then filled with Fuji IX GP (GC) to provide 1-year in vivo data. A year later, two more teeth in each monkey were similarly prepared and restored for the 1-day in vivo group. The following day, the restored teeth were extracted and the restoration interfaces observed using transmission electron microscopy (TEM). In addition, restorations were similarly placed in two extracted human teeth (control, 1-day in vitro group) and observed a day after placement using TEM.

**Results:**

The 1-day in vivo and in vitro results showed that the GIC appeared to bond to dentin through a demineralized zone similar to the hybrid layer produced by resinous adhesives. However, the interface between GIC and dentin after 1 year in vivo appeared to change over time: many needle-like crystals were detected within the remineralized layer and along the collagen fibrils. Slow diffusion of ions resulted in pores, which filled with mineral crystals and made the pores smaller.

**Conclusion:**

The interface between GIC and dentin morphologically changes over time, and recrystallization or remineralization at the interface may occur (1 year in vivo).

Glass-ionomer cements (GICs) were developed in the early 1970s.^36^ Conventional GICs have several advantages over other restorative materials. They release fluoride ions over the long term, which have caries preventive properties and help remineralize adjacent tooth tissues.^19,20^

The adhesion mechanism between GICs and dentin is based on an ion-exchange process.^15-17,19,20^ Polyacrylic acid demineralizes the superficial tooth surface, and subsequently, a strong ionic bond between the calcium of the hydroxyapatite and the carboxyl groups of the polyacrylic molecules is formed, as reported using x-ray photo-electron spectroscopy.^39^

Regarding clinical effectiveness, it was reported that GICs bond to cervical noncarious class-V lesions most effectively and durably.^23^ In addition, GICs showed superior clinical survival rates for deep dentin and hypermineralized dentin.^22,32^

The traditional concept of caries removal is changing from full caries removal to partial caries removal or incomplete caries removal using stepwise removal and selective removal techniques based on scientific evidence.^2,11,24^ For these situations, GICs should be recommended for use, as they show similar bond strengths to both normal and caries-affected dentin, in addition to having anti-bacterial and remineralizing effects.^4,14,18,31^

However, there is little information on the durability or interfacial ultrastructure of conventional GICs, although data have been reported for resin-modified GICs.^33,40^

This purpose of this study was to morphologically evaluate the interface between a conventional GIC and dentin 1 day after placement, as well as after functioning for 1 year in vivo, using transmission electron microscopy (TEM). The study was designed to be qualitative in nature.

## Materials and Methods

The materials used in this study are shown in Table 1. The experimental design of the animal study was approved by the ethics committee of Hokkaido University, and the experiments were performed under the regulations of the Institute of Laboratory Animals, Hokkaido University. The study design using monkeys followed previous published work on interfaces.^12^ Two monkeys (*Macaca fascicularis*) were placed under general anesthesia by intramuscular injection of 22 mg/kg ketamine (Veterinary Ketalar 50, Sankyo; Tokyo, Japan). Standardized shallow saucer-shaped class V dentin preparations, 1.5 mm deep and half the width of the tooth’s facial surface, were prepared on the facial surfaces of 7 intact teeth in each monkey to provide a total of 14 (maxillary and mandibular) teeth, using a high-speed tapered diamond bur (440, GC; Tokyo, Japan) under water spray. The bur was replaced after each preparation. All cavosurface margins were in enamel, but the enamel was thinner near the gingival extension. The cavities were conditioned using Cavity Conditioner (GC), and then restored with a highly viscous conventional GIC (Fuji IX GP, GC). These restorations were referred to as the 1-year in vivo group. One year later, two other teeth in each monkey were restored in vivo using the same methods and materials; these teeth were the 1-day in vivo group. Both monkeys were then sacrificed by injection of 125 mg/kg suxamethonium chloride (Succin, Yamanouchi Pharmaceutical; Tokyo, Japan) and 3.8 mg/kg thiopental sodium (Ravonal, Tanabe Seiyaku; Osaka, Japan) one day after placement of the 1-day restorations. All restored teeth were immediately extracted and processed for transmission electron microscope (TEM) observation.

**Table 1:** The materials used in this study

Product name	Composition
Cavity Conditioner (GC; Tokyo, Japan)	20% polyacrylic acid, distilled water, aluminum chloride hydrate, food additive Blue No. 1
Fuji IX GP (GC)	Polyacrylic acid, aluminosilicate glass, proprietary ingredient

Additionally, to investigate the difference between the interfaces in vivo and in vitro, two extracted human third molars were used. They were used within 1 month after extraction and were restored in the same manner as mentioned above, before being stored in distilled water for 1 day. These comprised the control group.

All TEM specimens were processed in accordance with common procedures of the non-demineralized method used for ultra-structural TEM of biological tissues. This included fixation overnight in 2.5% glutaraldehyde in 0.1 M sodium cacodylate buffer at pH 7.4 and 4°C, hhen rinsing in 0.1 M sodium cacodylate buffer for 1 min with 3 changes. Subsequently, specimens were dehydrated in ascending grades of ethanol (50%, 75%, 95%, and 100%) for 10 min each, with 2 changes, and were then immersed in 1:1 absolute ethanol-epoxy embedding resin (Epon 812, Polysciences; Warrington, PA, USA) for 4 h. This was followed by resin-infiltration in 100% epoxy embedding resin for another 8 h, and finally, embedding in molds with 100% epoxy resin. After embedding, epoxy blocks were polymerized in an oven at 60°C for 48 h.

Ultrathin sections (70-90 nm thick) through the fractured plane were cut using a diamond knife (Diatome; Bienne, Switzerland) in an ultramicrotome (Ultracut UCT, Leica; Vienna, Austria). The ultrathin sections were observed with a TEM (H-800, Hitachi; Tokyo, Japan) operating at 75 kV.

## Results

**Fig 1:**
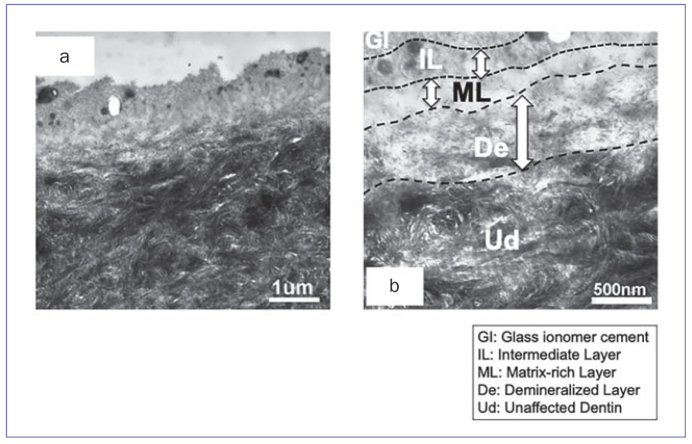
Non-demineralized, unstained TEM photomicrographs of the interface between dentin and GIC of a 1-day in vitro (control) sample. a: 25,000X magnification of the interface: the superficial dentin zone towards the GIC appeared to be demineralized almost completely. Collagen fibrils were observed within this zone due to the electron density of the interfibrillar spaces being higher than that of the collagen fibrils. b: 50,000X magnification of the interface: the thickness of the demineralized layer (De) was approximately 1 μm. Above the demineralized layer (De), a matrix-rich layer (ML) was seen. On top of the ML layer, an intermediate layer (IL) of a few hundred nanometers was noted. GI: glass-ionomer cement; IL: intermediate layer; ML: matrix-rich layer; De: demineralized layer; UD: unaffected dentin.

**Fig 2:**
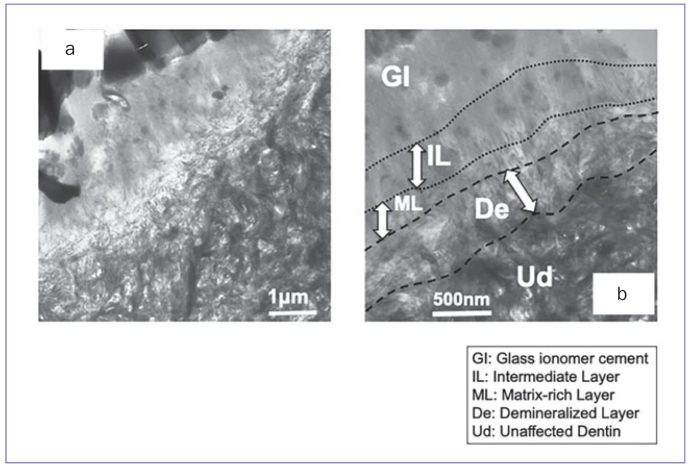
Non-demineralized, unstained TEM photomicrographs of the interface between dentin and GIC of a 1-day in vivo sample. a: 25,000X magnification of the interface: compared to the image of a control specimen (Fig 1a), the hydroxyapatite crystals were not completely dissolved at the interface. In addition, the number of black globules was less than in the image of a control specimen (Fig 1a). b: 50,000X magnification of the interface: denuded collagen fibrils at the interface were not clearly observed, although some denuded collagen fibrils could be observed at lower magnification (Fig 2a). Residual hydroxyapatite crystals were visible at the interface. The thickness of the demineralized layer with a significant amount of residual hydroxyapatite crystals was approximately 500 nm, which appeared thinner than that of the control (Fig 1b). In addition, many small black globules, which were hardly detected in the lower magnification image, were observed within the intermediate layer (IL). GI: gIass-ionomer cement; IL: Intermediate Layer; ML: matrix-rich layer; De: demineralized layer; UD: unaffected dentin.

TEM images of the control (Fig 1) and 1-day in vivo specimens (Fig 2) revealed that the superficial dentin zone was partially demineralized to a depth of about 500 nm to 1 µm, leaving hydroxyapatite crystals in the middle and at the bottom of this zone. The width of the demineralized layer (De) in the control specimens (Fig 1a) was thicker than that of the 1-day in vivo specimens (Fig 2a). On top of the demineralized layer (De), a matrix-rich layer (ML) was seen; this appeared to be of a few hundred nanometers thick (Fig 1b and 2b). On top of the matrix-rich layer (ML), an intermediate layer (IL) a few hundred nanometers thick was noted (Fig 1b and 2b).

**Fig 3:**
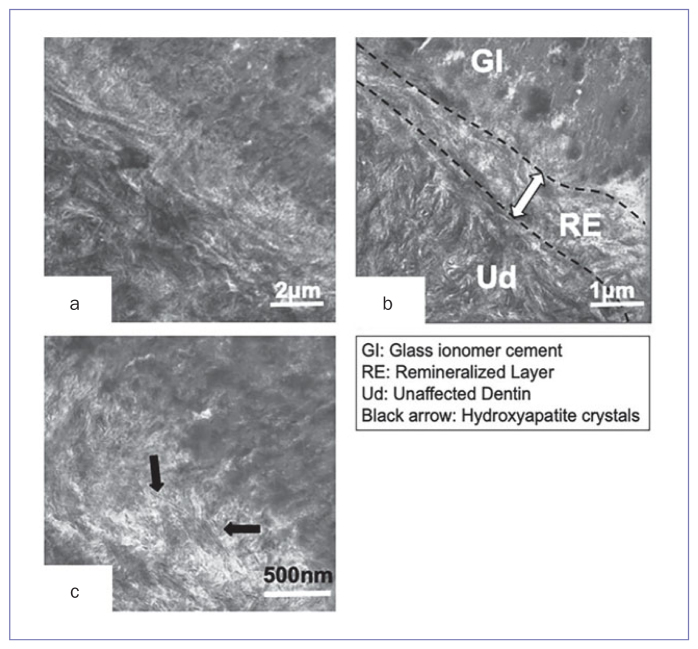
Non-demineralized, unstained TEM photomicrographs of the interface between dentin and GIC of a typical 1-year in vivo sample. a: 15,000X magnificationof the interface: black globules and demineralized layer, as were observed in Figs 1 and 2, could not be seen here. The electron density of GIC was as dense as that of the intact dentin zone. Naked collagen fibrils were not detected. Instead of the existence of black globules, many electron-dense spots were observed within the GIC matrix. b: 25,000X magnification of the interface from another tooth specimen: although the collagen fibrils coated by crystals were clearly detected, denuded collagen could hardly be observed. In the GIC matrix, electron-dense structures, which consisted of the aggregation of black spots, were observed. The thickness of the remineralized layer (RE) was approximately 700 nm to 1μm. c: 50,000X magnification of Fig 3b specimen: within the remineralized layer (RE), many crystals were seen. GI: glass-ionomer cement; RE: remineralized layer; UD: unaffected dentin. Black arrow: hydroxyapatite crystals.

In contrast, for the 1-year in vivo group (Fig 3a), the electron density of the GIC matrix was as dense as that of the intact dentin zone. The demineralized layer as observed in the control and 1-day in vivo specimens (Figs 1 and 2) was not seen. Instead, on top of the unaffected dentin (UD), the zone seemed remineralized because the electron density of this zone was as dense as that of the intact dentin zone. The thickness of the remineralizing layer (RE) was approximately 500-700 nm (Figs 3a and 3b). At higher magnification (Fig 3c), many hydroxyapatite crystals were clearly observed within the interface.

## Discussion

This is the first report on the long-term in vivo morphological changes of the interface between a conventional GIC and dentin using TEM methodology. Previously, our laboratory reported the durability of the interfaces between adhesive resins and dentin after 1 year of functioning in a monkey’s mouth, using a microtensile bond strength (µTBS) test and two kinds of electron microscopic observations (FE-SEM and TEM).^12,13^ The results showed that the adhesive interfaces produced by an etch-and-rinse adhesive was less resistant to degradation, as indicated by the gradually decreasing stainability of the hybrid layer, widened interfibrillar spaces, and the rather loosely organized collagen network along the dentinal tubule wall, compared to that produced by a mild self-etch adhesive. Especially in morphological observations, we detected signs of degradation of collagen fibrils within and at the bottom of the hybrid layer, when using the etch-and-rinse adhesive. This may be due to excessive acid etching of the superficial dentin by phosphoric acid, inadequate penetration of resin monomer into the demineralized dentin zone, and/or inadequate in situ polymerization of resin. Moreover, for both adhesives, the interface between the adhesive resin and a resin composite was also degraded.

Conventional GICs offer several advantages over other restorative materials; however, the bond strength between GIC and dentin has been reported to be lower than that of resinous materials.^8,37^ The relative setting shrinkage of GIC is zero, which is partially due to expansion from water sorption at high enough moisture levels, such as are found in the mouth.^1,3^ The setting stress of GIC is much lower than that of resinous materials. Hence, this material does not need high bonding capacity to tooth structure. Moreover, according to fractographic analysis of bond breakage between GIC and dentin, most fractures occur cohesively within GIC.^8,37^ This means that the low bond strength could be due to the brittleness of GICs.

There are very few TEM studies of GICs or glass-ionomer–based adhesives in the literature.^8,29,30,33,37,38^ This may be due to the difficulties in preparing and sectioning GIC-dentin specimens for TEM observation. In the present study, we used non-demineralized and unstained specimens, because the polyalkenoate matrices and glass particles of GICs are soluble in the acidic solvent used for the laboratory demineralization and staining of thin sections.

In the 1-day in vivo and control groups (Figs 1 and 2), a demineralized layer was observed at the interface, similar to the hybrid layer, which is produced by mild self-etching adhesives.^33^ When GIC is placed into a cavity, the superficial dentin and the smear layer are demineralized by the polyacrylic acid conditioner and the acidity of the GIC itself.^10,26^ That is why a demineralized layer is created. The use of polyacrylic acid conditioner is recommended in terms of bonding interface quality and durability.^7^ Polyacrylic acid, contained in the conditioner and GIC liquid, can only penetrate into the demineralized layer to a depth of less than 1 to 2 µm, due to its hydrophilicity and viscosity.^30^ In the 1-day in vivo and control groups (Figs 1 and 2), the electron density of collagen interfibrillar spaces was similar to that of the GIC matrix. The width of this demineralized layer in control specimens (almost 1 µm) was wider than that of the 1-day in vivo specimens (almost 500 nm) (Figs 1a and 2a). This may be due to the fact that the conditioner could not work adequately in the 1-day in vivo specimens, because remaining water was contained in the superficial dentin and smear layer, and exudates from the dentinal tubules were present. The in vivo dentin surface is moister than the in vitro dentin surface. This means that dentin of an in vivo tooth has greater buffering ability against the acid attack by a polyacrylic conditioner and GIC itself. Hence, the amount of reactive metallic ions from dissolved hydroxyapatite crystals of the in vivo condition was less than that of the in vitro condition. Additionally, as previously reported, on top of the demineralized layer (De), a matrix-rich layer (ML) and an intermediate layer (IL) were observed for both the in vitro and in vivo specimens.^11^

After functioning in the oral environment for 1 year, the features of the demineralized layer were altered (Fig 3). The electron density of this layer was higher than that of the control and the 1-day in vivo groups. At higher magnification, in 1-year in vivo specimens, almost all collagen fibrils within the demineralized layer were coated by crystals (Fig 3c). Moreover, the collagen interfibrillar space, which was observed in the control and the 1-day in vivo groups, was not seen; rather, needle-like crystals were observed within the interface. This must be attributed to the GIC, as a previous study, which reported 1-year in vivo results using resin composite materials, reporte a completely different appearance: there was no remineralized layer at all.^12^

One study observed increased mineralization (Ca, P) in the axial wall of surface dentin to a depth of about 20 µm 30 days (with water immersion) after a conventional GIC had been applied.^9^ To date, several studies have demonstrated evidence of the existence of an intermediate layer along the GIC-dentin/enamel interface, which is caused by ion exchange between the material and tooth substrate.^5,6,25,30,37^ An intermediate layer or ion-exchange layer is created by diffusion, in which ions from both the cement and the tooth move into the interfacial area, which results in strong adhesion between the cement and the tooth.^21,27^ This exchange of ions reacts slowly, which results in durable bonds and prevents leakage into the underlying natural tooth.^21^

Water permeation from dentin into conventional GICs or resin-modified GICs has been observed.^28,34,35^ As vital teeth were used in the present study, the interfacial dentin zone had an abundance of metallic ions delivered from the pulp through the dentinal tubules.

In remineralization and/or recrystallization, Ca and P ions are necessary for the crystals to grow. At the interface of the 1-day in vivo and control specimens, an electron-lucent demineralized layer was observed (Figs 1 and 2). This means that the hydroxyapatite crystals were destroyed, dissolved, and extracted by the polyacrylic acid conditioner or the acidity of the GIC itself, and Ca and P ions were separated. As the electron density of these ions was low, we could not observe them. After 1 year, however, many needle-like crystals were detected within the remineralized layer and along the collagen fibrils at the interface (Fig 3). Our speculation is that various ions from GIC, the dissolved hydroxyapatite crystals (as stated above), dentinal tubules and/or the oral environment may be related to the formation of polyacid salts and help crystals remineralize over time. Slow diffusion of ions may affect the demineralized area such that it is filled with mineral crystals, making the pores smaller.

## Conclusion

This study showed that 1. conventional GIC bonds to dentin through a demineralized layer similar to the hybrid layer produced by resinous adhesives during the initial phase (1 day after filling); 2. the interface between GIC and dentin changes over time (1 year after filling); and 3. the possibility of recrystallization or remineralization at the interface occurs morphologically (1 year after filling).

## Clinical relevance:

The interface between GIC and dentin changes over time and the possibility of recrystallization or remineralization at the interface occurs morphologically compared with resin composite materials.
